# Detergents without a drain: the evolutionary logic (and liability) of sphingolipids

**DOI:** 10.1016/j.jlr.2026.100998

**Published:** 2026-02-13

**Authors:** Scott A. Summers

**Affiliations:** Department of Nutrition and Integrative Physiology, University of Utah, Salt Lake City, UT, USA

**Keywords:** lipotoxicity, ceramides, sphingolipids, evolution, membrane biophysics, cardiovascular disease, diabetes

## Abstract

Sphingolipids are evolutionarily conserved lipids that, I contend, emerged as a solution to a fundamental biochemical problem: cells require fatty acids, yet these molecules are potent detergents. In higher metazoans, a metabolic asymmetry amplifies this physical threat: unlike most macronutrients, fatty acids cannot be readily converted into nonlipid forms of biomass. Thus, when their supply exceeds energetic demand, they remain chemically committed lipids with the capacity to destabilize membranes and disrupt cellular organization. The emergence of sphingolipid metabolism offered an elegant solution to this challenge. By incorporating fatty acids into sphingolipids, cells both stabilize membranes to combat detergent stress and generate ceramide-dependent signaling programs that coordinate metabolic adaptation, remodeling, and, when necessary, cell elimination in response to lipid overload. In modern settings of chronic lipid surplus, most prominently obesity, this otherwise adaptive system becomes pathological. Across liver, adipose tissue, skeletal muscle, heart, pancreas, and kidney, excessive sphingolipid accumulation enforces metabolic inflexibility, impairs mitochondrial efficiency, and promotes cell dysfunction or loss, contributing to diabetes, steatohepatitis, heart failure, and kidney disease. Human studies consistently associate circulating ceramide species with cardiometabolic risk, while interventional studies in rodents demonstrate their causal roles in disease progression. Together, these findings position sphingolipids—much like cholesterol—as both early biomarkers and modifiable drivers of chronic disease, highlighting how an evolutionary solution becomes pathogenic in the setting of prolonged nutrient excess.

Life’s earliest membranes faced a basic challenge: how to coexist with fatty acids—molecules that were both essential and dangerous. Saturated fatty acids, in particular, are chemically simple yet biophysically hazardous. When present as unesterified amphiphiles, their detergent-like properties can destabilize lipid bilayers, increasing permeability and disrupting membrane organization. Moreover, their metabolism presents a second, more insidious challenge: unlike anaplerotic nutrients such as glucose or amino acids, fatty acids cannot be readily converted into proteins, nucleotides, or other forms of biomass (1). When their intake or synthesis outpaces energetic demand, this metabolic inflexibility creates a bottleneck—leaving cells with excess lipid derivatives that must be buffered, sequestered, or transformed.

Prokaryotes stabilize their membranes using lipid chemistries distinct from those found in eukaryotes—including hopanoids, branched or cyclopropanated fatty acids, and, in archaea, ether-linked isoprenoids. These strategies effectively modulate bilayer packing and permeability in organisms that retain a rigid cell wall, but are poorly suited to membranes that must support rapid curvature, vesicle budding, fusion, and protein-dense signaling platforms. In such systems, fatty acids are frequently liberated—both through lipolysis during nutrient flux and through phospholipid turnover accompanying membrane remodeling. When present at sufficiently high concentrations, these amphipathic lipids can introduce packing defects or detergent-like perturbations (2). In this context, sphingolipids emerged and, together with sterols, provided a new membrane architecture capable of tight packing, low permeability, and resistance to fatty acid–induced disordering—a strategy distinct from the stabilizing mechanisms retained by most prokaryotes (3).

Sphingolipids—particularly ceramides—also came to serve as signals of lipid excess, triggering coordinated metabolic and stress-adaptive responses. For example, ceramides act as bioactive messengers that suppress glucose uptake, reprogram mitochondrial metabolism, and, under prolonged stress, trigger apoptosis. These responses were likely advantageous in fluctuating nutrient environments, where transient lipid surges demanded swift reorganization of cellular priorities. But in modern settings of chronic overnutrition and sustained fatty acid availability, ceramide levels rise persistently—reflecting increased substrate flux through the sphingolipid biosynthetic pathway. Under these conditions, the very properties that once protected cells become liabilities: membrane rigidity impairs fluidity and protein mobility, while prolonged signaling enforces maladaptive metabolic reprogramming and eventual cell death (4).

This perspective has been shaped not only by experiments in our lab, but by years spent grappling with a simple, recurring observation: sphingolipids drive many of the cellular adaptations that underlie cardiometabolic disease (5). The opportunity to deliver the *Journal of Lipid Research* Lectureship at the Keystone Symposium on Lipids in Cellular Function and Disease offered a welcome chance to reflect on how our field got here—both scientifically and evolutionarily. In this essay, I aim to trace the arc of sphingolipid biology, from its emergence as a membrane-stabilizing innovation to its entrenchment in the pathogenesis of diabetes, metabolic dysfunction–associated steatohepatitis (MASH), heart failure, and end stage renal disease. Along the way, I will explore the idea that lipid evolution, like metabolic signaling itself, is a story of adaptation and eventual maladaptation.

## The problem with fatty acids

Long before cells evolved the intricate lipidomes we recognize today, they faced a physicochemical paradox: fatty acids are vital sources of energy and membrane components, yet intrinsically disruptive when left unregulated. In their unesterified form, long-chain fatty acids readily partition into lipid bilayers and undergo rapid transbilayer movement, driven by their amphipathic nature. This diffusional capacity allows for passive nutrient uptake (6, 7, 8, 9, 10). But when present at higher local concentrations—such as during fasting, overnutrition, or inflammatory stress—these fatty acids can disrupt bilayer packing, alter membrane fluidity, and compromise protein function (11).

As an early response to this challenge, the first prokaryotes relied on vectorial acylation and CoA esterification as the foundational mechanisms for managing unesterified fatty acids. Acyl-CoA synthetases activate fatty acids in a directional, membrane-associated manner, neutralizing their detergent-like behavior and committing them to downstream pathways including glycerophospholipid synthesis and β-oxidation (12, 13). These systems constitute the ancestral machinery for fatty acid handling and remain the dominant means by which modern cells regulate fatty acid flux.

To further manage the disruptive potential of unesterified fatty acids, early prokaryotes evolved strategies to sequester these molecules in more inert, membrane-compatible forms (Table 1). They channeled the fatty acid chain from acyl-CoAs into the biosynthesis of glycerophospholipids through the stepwise action of enzymes such as glycerol-3-phosphate acyltransferase, lysophosphatidic acid acyltransferase, and diacylglycerol acyltransferase (DGAT)—all of which originated in prokaryotes and sequentially esterify fatty acids to a glycerol backbone (14).

**Table 1:** Timeline denoting approximate evolutionary appearance of key lipid metabolism gene families

Evolutionary Timing (Approximate)	Module/Enzyme Family	Representative Genes
Prokaryotic	Acyl-CoA synthetases (ACSLs)	*ACSL1–6*
Prokaryotic–early eukaryotic	Glycerolipid-synthesizing enzymes	*GPAT1–4*, *AGPAT1–10*, *DGAT1/2*
Prokaryotic–eukaryotic	Phospholipases (PLA, PLC, PLD families)	*PLA2G4A*, *PLCB1–4*, *PLD1/2*
Unicellular eukaryotes	FATPs (SLC27 family)	*SLC27A1–6 (FATP1–6)*
Bacteria–lower eukaryotes	Medium-chain acyl-CoA synthetases (ACSMs)	*ACSM1–6*, *SLC27A5 (FATP5)*
Early eukaryotic	Sphingolipid-synthesizing enzymes	*SPTLC1/2*, *CERS1–6*, *DEGS1*, *SGMS1/2*
Early eukaryotic	Sterol biosynthesis (e.g., HMG-CoA Reductase)	*HMGCR*, *SQLE*, *DHCR7*, *CYP51A1*, *LSS*
Metazoan	FABPs (fatty acid–binding proteins)	*FABP1–9*
Metazoan	Lipid droplet biogenesis proteins	*PLIN1–5*, *FIT1/2*, *CIDEC (FSP27)*
Vertebrate	CD36 (fatty acid translocase)	*CD36*

Gene modules involved in lipid activation, synthesis, remodeling, transport, and storage are shown in approximate order of appearance based on comparative genomics, phylogenetic inference, and database annotations. The evolutionary timing reflects the first emergence of homologs in major taxonomic groups: prokaryotes, lower eukaryotes, early multicellular organisms (metazoans), or vertebrates. Core genes associated with each module are listed, including acyl-CoA synthetases (ACSLs, ACSMs), glycerolipid- and sphingolipid-synthesizing enzymes (e.g., GPATs, CERS, SGMS), phospholipases, fatty acid transporters (FATPs, CD36), lipid droplet proteins (PLINs, CIDEC), sterol biosynthesis genes (e.g., HMGCR, SQLE), and fatty acid chaperones (FABPs). Gene family emergence was cross-referenced against multiple databases—including NCBI HomoloGene, KEGG Orthology, and OrthoDB—and confirmed using the Gene CLiME (Clustering by Inferred Models of Evolution) resource developed by the Mootha laboratory, which infers evolutionary modules based on gene co-retention across eukaryotic lineages.

DGAT, diacylglycerol acyltransferase; FATP, fatty acid transport protein; GPAT, glycerol-3-phosphate acyltransferase.

Eukaryotes inherited and expanded these systems, elaborating them into specialized, compartmentalized pathways that supported the synthesis and remodeling of diverse lipid species. This evolutionary step allowed for the development of dynamic phospholipid bilayers, laying the groundwork for the flexible and internally complex membranes that characterize eukaryotic cells. Eukaryotes further evolved sphingolipid biosynthesis enzymes, introducing saturated, long-chain lipids that paired with sterols to form raft domains—conferring detergent-resistant properties to these emerging bilayers (15, 16).

To safely store excess lipids, lipid droplet–associated proteins also emerged (17, 18), enabling the packaging of neutral lipids into organelles surrounded by a phospholipid monolayer and regulated by coat proteins such as perilipins. Moreover, multicellular organisms expanded and diversified the lipid-handling machinery that was emerging in unicellular organisms—including the specialized fatty acid transporters such as CD36, and increasingly complex families of fatty acid transport proteins and fatty acid–binding proteins. These systems allow for more tightly regulated uptake and intracellular trafficking of fatty acids across tissues. By accelerating the conversion and compartmentalization of free fatty acids, they help buffer membranes from the detergent-like effects of lipid overload.

Each stage of this progression reflects a strategy of molecular sequestration—embedding fatty acids within structural hierarchies to mitigate their cytotoxicity while expanding cellular metabolic capacity.

Despite the emergence of increasingly sophisticated lipid-handling machinery, one feature of vertebrate fatty acid metabolism remains puzzling: lipids are generally not anaplerotic. When fully oxidized, even-carbon fatty acids generate acetyl-CoA, which enters the tricarboxylic acid (TCA) cycle but does not replenish it. For each two-carbon unit that enters via acetyl-CoA, two carbons are lost as CO_2_—yielding no net gain in TCA intermediates. By contrast, pyruvate and several amino acids contribute carbon directly to the cycle’s intermediates, supporting both energy production and the biosynthesis of different macromolecular classes. This characteristic renders fatty acids a dense fuel for ATP generation but metabolically inflexible—they cannot support the net synthesis of proteins, nucleotides, or other biomass components.

Through a bypass of the decarboxylation steps of the TCA cycle, plants, fungi, and many prokaryotes use the glyoxylate shunt to redirect lipid-derived acetyl-CoA into four-carbon biosynthetic intermediates such as malate and succinate, enabling their conversion into glucose, amino acids, and other anabolic products (19). Vertebrates lack this pathway, eliminating a major route for reclaiming lipid carbon for anabolism and confining fatty acid oxidation largely to energy production. As a result, lipid surplus cannot be dissipated into nonlipid biomass, increasing the pressure on eukaryotic cells to buffer or sequester excess fatty acids—particularly during lipolysis, when membrane integrity is most vulnerable and alternative disposal routes are limited.

This asymmetry created pressure for new lipid-handling systems. They needed to neutralize the disruptive physical properties of fatty acids while also managing their metabolic surplus. Sphingolipids emerged within this evolutionary context as multifunctional molecules that incorporated excess fatty acids, conferred detergent resistance to the membrane bilayer, and served as signals of lipid excess that reprogrammed cellular metabolism to accommodate potential surpluses of dangerous fatty acids.

## The architecture of a solution

**Fig. 1:**
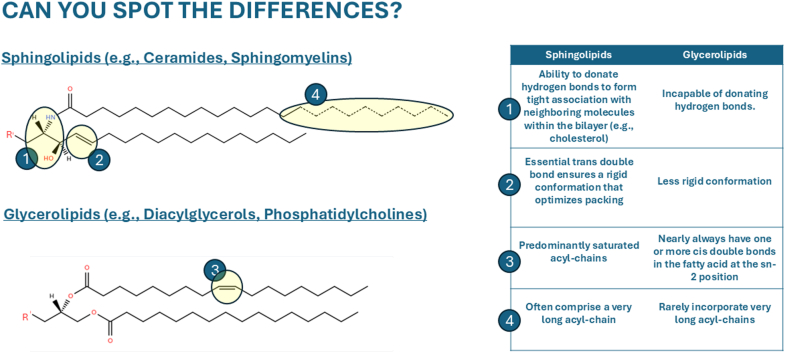
Structural features of sphingolipids promote membrane order and domain formation. Sphingolipids possess long, saturated acyl chains and amide-linked head groups that promote tight packing and reduced membrane fluidity. These attributes allow them to associate with cholesterol, which facilitates the formation of lateral membrane domains (rafts) that confer detergent resistance to membranes and serve as organizing centers for signaling and trafficking.

To understand how sphingolipids solved the biophysical and metabolic liabilities posed by excess fatty acids, one must first consider their molecular architecture. The distinctive chemistry of sphingolipids—rather than their abundance—underlies their capacity to stabilize membranes, resist detergent stress, and form ordered domains. These structural features set sphingolipids apart from the glycerolipids that dominate most biological membranes (20, 21) (Fig. 1).

Let us begin by considering the basic structure of the glycerolipids, which are both far more abundant and evolutionarily older than sphingolipids. Built on a glycerol backbone that accommodates up to three esterified acyl chains, glycerolipids serve as the principal cellular sink for fatty acids. However, the glycerol scaffold functions exclusively as a hydrogen-bond acceptor and cannot donate hydrogen bonds, limiting its ability to form tight, cooperative intermolecular interactions within lipid bilayers (20, 21). In addition, glycerolipids almost invariably incorporate an unsaturated—often polyunsaturated—fatty acyl chain at the sn-2 position (22). These unsaturations promote bilayer fluidity, facilitate lateral diffusion of membrane proteins, and support dynamic membrane remodeling. However, they also disrupt acyl-chain packing, introduce conformational disorder, and lower bilayer melting temperature (23, 24).

By contrast, the defining feature of sphingolipids is a nitrogenous, amino acid–derived sphingoid backbone capable of donating hydrogen bonds and engaging in extensive intermolecular interactions (20, 21). This backbone architecture, together with the relative paucity of strategically disruptive unsaturation, promotes tight molecular packing and elevated bilayer order. With the exception of a conserved *trans* double bond within the sphingoid backbone—which, paradoxically, enhances rather than disrupts packing and is discussed in detail below—sphingolipids generally lack unsaturation at positions that would destabilize bilayer structure. Even in the subset of ceramides that contain a single unsaturation (e.g., C18:1 or C24:1), the double bond lies deep within the acyl-chain tail (typically at the Δ15 position), where it minimally perturbs packing (25). Together, these structural differences underscore the distinctive capacity of sphingolipids to promote membrane order relative to glycerolipids.

**Fig. 2:**
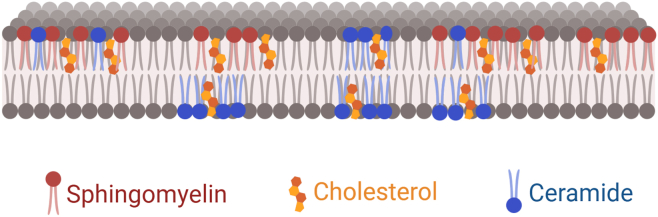
Lipid organization within the plasma membrane. Sphingomyelin (SM) and ceramide cluster with cholesterol to form lateral membrane platforms, often termed lipid rafts. These domains modulate membrane structure and signaling. SM is enriched in the exofacial (outer) leaflet, while ceramide can insert into both leaflets and disrupt membrane order. This illustration is a conceptual cartoon and does not reflect molecular scale or dynamics.

As a consequence of this architecture, sphingolipids assemble into highly ordered microdomains. These properties become especially significant when such domains cluster—often in conjunction with cholesterol—into high-melting temperature, detergent-resistant regions that stabilize bilayers under thermal, biophysical, and chemical stress (Fig. 2). Tight intermolecular associations enhance lateral cohesion within the membrane, reducing susceptibility to solubilization by amphipathic agents and preserving membrane integrity under hostile conditions.

Consistent with these biophysical properties, sphingolipid metabolism is dynamically regulated by thermal stress across a wide range of organisms. Heat shock rapidly induces sphingoid base and ceramide synthesis in yeast (26, 27) and activates de novo ceramide production in mammalian cells (28). More recent work extends these observations across phylogeny: acute thermal stress drives coordinated remodeling of membrane lipids—including sphingolipid classes—in oysters, influencing temperature adaptation (29), while sphingolipid signaling also participates in cnidarian heat-stress responses (30). Temperature shifts similarly regulate sphingolipid desaturation pathways during cold stress in plants (31). In humans, a recent panel study reported associations between nonoptimal temperature exposure and markers of sphingolipid metabolism, including ceramide species (32). Together, these findings support the idea that sphingolipids constitute part of a conserved cellular response to membrane-destabilizing forces, as evidenced by their enrichment under temperature fluctuations that challenge bilayer integrity.

## Installing the architecture: sphingolipid biosynthesis and diversification

**Fig. 3:**
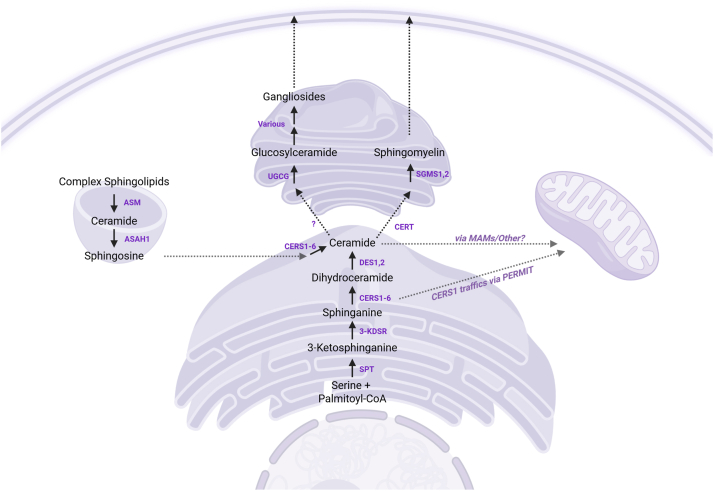
Subcellular topology of sphingolipid biosynthesis and trafficking. Sphingolipid synthesis initiates in the endoplasmic reticulum (ER), where serine and palmitoyl-CoA condense to form 3-ketosphinganine. Sequential enzymatic steps produce dihydroceramide and ceramide, which is either retained in the ER or trafficked to the Golgi via a ceramide transfer protein (CERT). In the Golgi, ceramide is converted into sphingomyelin, glucosylceramide, and complex gangliosides. Ceramide can also be routed to mitochondria—potentially via mitochondria-associated membranes (MAMs)—or metabolized back into sphingosine through lysosomal degradation. Abbreviated enzymes include the following: (1) Serine palmitoyltransferase (SPT): catalyzes the first step in sphingolipid synthesis by condensing serine and palmitoyl-CoA; comprises SPTLC1, SPTLC2, and SPTLC3 subunits. (1) 3-ketodihydrosphingosine reductase (3-KDSR): reduces 3-ketosphinganine to sphinganine. (2) Ceramide synthases (CERS): A family of enzymes that acylate sphinganine or sphingosine to form dihydroceramides or ceramides; isoforms include CERS1, CERS2, CERS3, CERS4, CERS5, and CERS6, which differ by tissue location and substrate (acyl-CoA) specificity. (3) Dihydroceramide desaturase (DES): converts dihydroceramide to ceramide by introducing a *trans* double bond; DES1 is the major isoform in most tissues, while DES2 is restricted to gut and skin. DES2 has additional hydroxylase activity, allowing it to generate the phytoceramides that are abundant in epithelial tissues. Gene names are DEGS1 and DEGS2, respectively. (4) Sphingomyelin synthases (SGMS): catalyze the transfer of a phosphocholine head group from phosphatidylcholine to ceramide, forming sphingomyelin; isoforms are SGMS1 and SGMS2. (5) UDP-glucose ceramide glucosyltransferase (UGCG): adds glucose to ceramide to generate glucosylceramide, the precursor of complex glycosphingolipids. (6) Acid ceramidase (ASAH1): hydrolyzes ceramide into sphingosine and a free fatty acid within lysosomes. Additional ceramidase isoforms reside in other locations within the cell and include ASAH2 (neutral ceramidase) and ACER1-3 (alkaline ceramidases). Some PAQR receptors (e.g., the adiponectin receptor) have been shown to have ligand-activated ceramidase activity. (7) Acid sphingomyelinase (ASM): degrades sphingomyelin into ceramide under acidic conditions; encoded by the SMPD1 gene. Other sphingomyelinases include the neutral sphingomyelinases SMPD2 and SMPD3, and the less well-characterized SMPD4 and SMPD5. (8) Ceramide transfer protein 1 (CERT1) transports ceramide from the ER to the Golgi apparatus for sphingomyelin synthesis. In addition, we highlight that MAMs facilitate the transfer of ceramides into mitochondria, but emphasize that this is an active and important area of investigation that is likely to uncover other regulatory mechanisms.

Sphingolipid biosynthesis begins with the condensation of serine with a saturated fatty acyl-CoA—typically palmitoyl-CoA (33) (Fig. 3). This reaction, catalyzed by serine palmitoyltransferase (SPT), forms 3-ketosphinganine and sets the foundation for a lipid backbone that is chemically distinct from the glycerolipids that dominate most biological membranes. The inclusion of serine introduces a nitrogen atom, endowing this “sphingoid motif” with unique properties, including the potential to donate hydrogen bonds and engage in molecular interactions not available to most glycerolipids (33).

Six (dihydro)ceramide synthases (CERS1-6), which show distinct tissue distributions and substrate specificities, transfer a fatty acid from acyl-CoA to the sphingoid base (34). The acyl chains of sphingolipids are characteristically saturated and extended, a combination that drives tight packing and elevates the melting temperature of membranes (21).

Another key feature of the sphingoid motif is a double bond inserted by the enzyme dihydroceramide desaturase-1 (DES1), which is present in nearly all tissues. DES1 inserts a critical *trans* double bond into dihydroceramides to produce ceramides, the major scaffold of complex sphingolipids. This conserved structural feature locks the backbone into a rigid conformation that optimizes internal hydrogen bonding, increases dipole potential, and enhances packing interactions with cholesterol—properties critical for the formation of ordered membrane domains (35). A related enzyme, DES2, instead introduces a hydroxyl group at the C4 position to generate phytoceramides, a class of sphingolipids enriched in skin, kidney, and the gut (36, 37). In contrast to ceramides, phytoceramides lack the *trans* double bond and are thus less rigid, with more limited roles in intracellular signaling. However, the additional hydroxyl group enhances their hydrogen-bonding capacity, promoting the formation of densely packed, water-impermeable barrier structures in epithelial membranes.

The insertion of diverse head groups at the C1 hydroxyl position further broadens the functional scope of these lipids. Among them, sphingomyelin stands out for its ability to colocalize with cholesterol in the exofacial leaflet of membrane bilayers, forming ordered membrane microdomains that confer detergent resistance and modulate protein distribution, signal transduction, and vesicle trafficking (15, 16). Other major derivatives include gangliosides, which carry complex carbohydrate chains and are enriched in the nervous system (38); ceramide-1-phosphate, a signaling lipid involved in inflammation and cell survival (39); and sphingosine-1-phosphate, a potent bioactive metabolite that regulates vascular integrity, immune cell trafficking, and proliferation (40, 41).

As sphingolipids confer biophysical stability and spatial organization to the bilayer, their accumulation—particularly in the form of ceramides—serves as a barometer of flux through the pathway. Elevated ceramide levels reflect increased availability of saturated fatty acids and serine, conditions often aligned with nutrient excess. In this way, the pathway doubles as both a buffer and a biosensor—transforming biochemical overflow into a signaling axis that links nutrient status to cellular fate.

## Ceramides: sentinels of lipid excess

Ceramides are not passive biosynthetic intermediates but active sentinels—molecules that both sense and respond to lipid surplus. At moderate levels, ceramides stabilize membranes, shift fuel preferences, and recalibrate mitochondrial efficiency. At higher concentrations, they enforce stricter thresholds—curtailing energy output and, if needed, initiating programmed cell death. Through these actions, ceramides act as metabolic gatekeepers: they help preserve cellular balance, but when overwhelmed, reprioritize metabolic processes or initiate apoptosis to protect the system as a whole.

### Membrane stabilization

Ceramides and sphingomyelins are uniquely suited to restore order in membranes disrupted by elevated fatty acid levels. Their saturated acyl chains and ability to form intermolecular hydrogen bonds enable tight packing within the bilayer, particularly in the outer leaflet of the plasma membrane. This stabilizing effect becomes especially important during states of lipid overload, where detergent-like fatty acids can fluidize membranes and compromise barrier function. By increasing bilayer cohesion and decreasing membrane permeability, ceramides act as structural buffers that reduce the disruptive impact of excess amphipathic lipids.

**Fig. 4:**
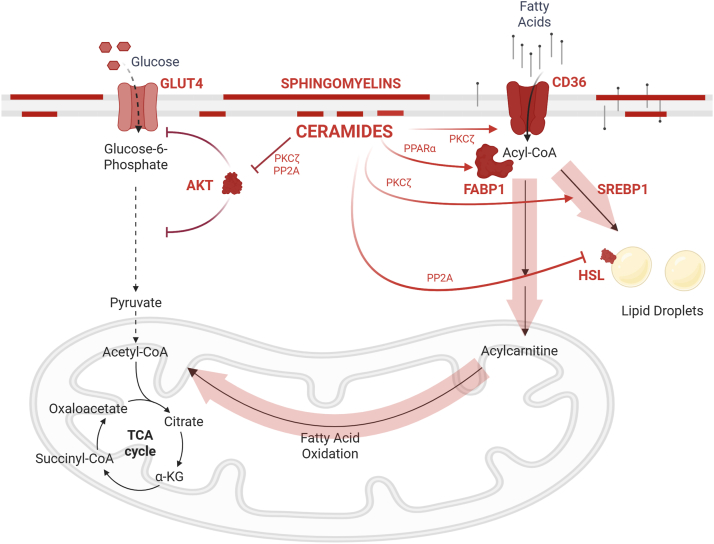
Ceramides shift cellular fuel preference toward lipids. Ceramides inhibit glucose uptake and utilization while promoting fatty acid uptake, storage, and oxidation. This reprogramming favors lipid-based metabolism at the expense of glucose-driven energy production, contributing to metabolic inflexibility. Abbreviations include the following: (1) AKT (protein kinase B): A central node in insulin signaling that promotes glucose uptake, glycogen synthesis, and cell survival. (2) Protein phosphatase 2A (PP2A): a serine/threonine phosphatase activated by ceramides; dephosphorylates and inactivates AKT. (3) (Protein kinase C-ζ (PKCζ): atypical PKC isoform activated by ceramides; impairs insulin signaling by interfering with AKT activation and GLUT4 translocation. (4) Peroxisome proliferator–activated receptor alpha (PPARα): a nuclear receptor that regulates genes involved in fatty acid oxidation, ketogenesis, and lipid transport; activated under fasting and lipid-rich conditions. Sphingolipids have been implicated as potential agonists. (5) Hormone-sensitive lipase (HSL): cytosolic lipase that translocates to lipid droplets to hydrolyze stored triacylglycerols into free fatty acids and glycerol; regulated by phosphorylation in response to catecholamines and insulin. (6) Sterol regulatory element-binding protein (SREBP): transcription factor family that governs lipid synthesis; SREBP1 drives fatty acid and triglyceride synthesis, while SREBP2 controls cholesterol biosynthesis. (7) Fatty acid–binding protein 1 (FABP1): cytosolic chaperone that binds long-chain fatty acids and facilitates their intracellular transport, especially in hepatocytes. (8) Carnitine palmitoyltransferase 1 (CPT1): rate-limiting enzyme in mitochondrial fatty acid import and oxidation; transfers acyl groups to carnitine at the outer mitochondrial membrane. (9) CD36 (fatty acid translocase): membrane protein that facilitates the transfer of long-chain fatty acids through the bilayer. (10) Glucose transporter type 4 (GLUT4): insulin-responsive glucose transporter in adipose tissue and skeletal muscle; ceramide accumulation impairs its translocation to the plasma membrane. (11) Tricarboxylic acid cycle (TCA cycle): central mitochondrial pathway that oxidizes acetyl-CoA to generate NADH, FADH2, and ATP precursors; replenished by anaplerotic substrates such as pyruvate and glutamine. (12) Alpha-ketoglutarate (α-KG): a TCA cycle intermediate produced from isocitrate and converted to succinyl-CoA.

An important parallel adaptation is that ceramides recruit CD36 (Fig. 4), a fatty acid translocase that facilitates the vectorial movement of fatty acids across bilayers, to the plasma membrane (42). By accelerating fatty acid uptake into cells, CD36 minimizes the residence time of unesterified fatty acids within the bilayer—thereby limiting their detergent-like effects and providing further stability to membrane structures. In this context, CD36 does not merely support metabolism, but serves as a defensive mechanism that protects membrane integrity under conditions of lipid excess. Ceramides also increase the expression of fatty acid–binding protein-1 (43), which chaperones fatty acids within cells.

### Altering fuel choice and storage

In parallel with their membrane-stabilizing effects, ceramides exert cell-autonomous control over nutrient utilization, reprogramming cellular metabolism to favor lipid handling over glucose metabolism (Fig. 4). A central action of ceramides is suppression of glucose uptake and glycolysis, mediated in part through inhibition of insulin and growth factor signaling to the proglycolytic kinase AKT via protein phosphatase 2A (PP2A) and atypical protein kinase Cζ (44, 45, 46, 47, 48, 49, 50, 51, 52). Concomitantly, ceramides promote increased reliance on fatty acids by enhancing fatty acid uptake and/or oxidation (42, 43) while simultaneously restraining lipid efflux by inhibiting lipolysis (53, 54) and favoring triglyceride (TG) storage (55). Through this coordinated shift—diverting glucose away from energy production while channeling fatty acids toward either oxidation or sequestration—ceramides align cellular metabolism with lipid-rich conditions characteristic of overnutrition.

At the organismal level, these same ceramide-driven mechanisms propagate insulin resistance across metabolic tissues, amplifying their cell-autonomous effects into a coordinated systemic program. Ceramides impair insulin sensitivity in muscle, adipose tissue, and liver, reducing insulin-stimulated glucose disposal and reinforcing reliance on lipid metabolism (53, 54, 55, 56, 57). In tandem, ceramides enhance storage of fatty acids as triacylglycerols through activation of lipogenic transcriptional programs (55) and inhibition of lipolytic enzymes (53, 54, 55), particularly in liver and adipose tissue.

These concerted actions not only enable the preferential use and sequestration of fatty acids, but also prevent harmful accumulation of unesterified lipids in membranes. While initially adaptive, this metabolic rerouting becomes maladaptive with chronic nutrient overload, contributing to “selective” insulin resistance (58), ectopic lipid deposition, and loss of metabolic flexibility.

### Tuning mitochondrial energy production

**Fig. 5:**
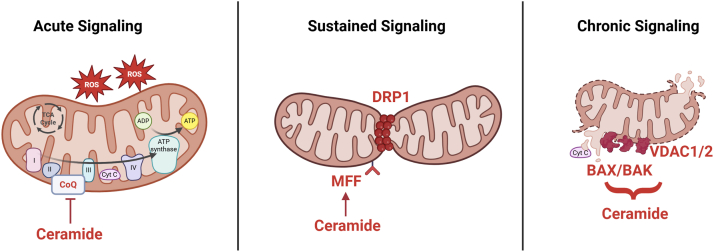
Progressive effects of ceramides on mitochondrial structure and function. Ceramides initiate a temporal cascade of mitochondrial stress responses, including acute effects on the electron transport chain (left), more sustained effects on mitochondrial fission leading to an overall decrease in size (middle), and terminal effects on mitochondrial outer membrane permeability that trigger apoptosis (right). Abbreviations include the following: (1) Coenzyme Q (CoQ, also known as ubiquinone). A lipid-soluble electron carrier that shuttles electrons between complexes I/II and III in the electron transport chain (ETC). (2) Cyt C: cytochrome c, a small heme protein that transfers electrons from complex III to complex IV in the ETC and plays a key role in apoptosis when released into the cytosol. (3) ATP synthase: ATP synthase complex (complex V). Uses the proton gradient across the inner mitochondrial membrane to synthesize ATP from ADP and inorganic phosphate. (4) ROS: reactive oxygen species. Byproducts of mitochondrial respiration, including superoxide, hydrogen peroxide, and hydroxyl radicals, especially elevated with ceramide accumulation. (5) TCA cycle: tricarboxylic acid cycle (6) DRP1: dynamin-related protein 1. A GTPase that mediates mitochondrial fission by constricting and dividing mitochondria. (7) MFF: mitochondrial fission factor. A receptor on the outer mitochondrial membrane that recruits DRP1 during fission events; ceramides enhance this interaction. (8) VDAC1/2: voltage-dependent anion channels 1 and 2. Outer mitochondrial membrane channels that regulate metabolite exchange and can form pores during apoptosis. (9) BAX/BAK: Bcl-2-associated X protein/Bcl-2 antagonist/killer. Proapoptotic proteins that oligomerize to permeabilize the outer mitochondrial membrane, enabling cytochrome c release.

Ceramides influence mitochondrial metabolism in complex and time-dependent ways that reflect adaptation to lipid excess rather than increased energetic demand. Under conditions of nutrient surplus, cellular ATP requirements are often already met, and continued maximal oxidative phosphorylation would risk excessive ATP production and reductive pressure within the electron transport chain. In this context, ceramide accumulation initially promotes fatty acid oxidation; however, this shift is accompanied by progressive changes that reduce oxidative phosphorylation efficiency, disrupt respiratory chain architecture, and increase oxidative stress (Fig. 5).

Mechanistically, ceramides impair oxidative phosphorylation efficiency by disrupting electron transport within the inner mitochondrial membrane. This may occur through displacement of coenzyme Q from the electron transport chain (59) or direct inhibition of electron transport chain components (60, 61, 62, 63), resulting in electron leak and increased production of reactive oxygen species (ROS). Ceramides generated by CERS6—which possess a 16-carbon acyl chain—also bind the mitochondrial fission factor (MFF), promoting mitochondrial fragmentation and impairing oxidative metabolism in adipose tissue and liver (64). Genetic or pharmacologic suppression of CERS6 preserves mitochondrial function, enhances insulin sensitivity, and improves whole-body energy expenditure under conditions of lipid excess. In addition to these effects, recent work has shown that ceramides disrupt the mitochondrial contact site and cristae organizing system, leading to altered cristae architecture and impaired respiratory efficiency (65). Notably, genetic or pharmacologic suppression of DES1 restores mitochondrial contact site and cristae organizing system integrity, normalizes mitochondrial structure, and improves oxidative metabolism under conditions of lipid excess, further supporting a causal role for ceramides in driving mitochondrial dysfunction. Collectively, these disruptions lower ATP yield per oxygen molecule and constrain maximal mitochondrial output, even as substrate oxidation persists.

As a result of these actions, ceramides drive the accumulation of ROS (66). These ROS damage mitochondrial membranes, proteins, and DNA, but also serve as a secondary route for eliminating excess reducing equivalents when the electron transport chain becomes saturated. In this way, ROS generation reflects both a damaging consequence and a compensatory mechanism for redox imbalance in lipid-stressed mitochondria.

### Receptor-mediated signaling

Recent studies have proposed a potentially important extension of ceramide biology by suggesting that select cell-surface receptors may mediate some of their biological effects. Beyond their established roles as intracellular messengers and structural lipids, ceramides have been reported to function as ligands for specific G-protein–coupled receptors, thereby directly engaging canonical signaling pathways. While this concept challenges traditional models of ceramide action, its broader relevance across tissues and disease contexts remains an open question.

Two G-protein–coupled receptors—CYSLTR2 and P2RY6—have been proposed as sensors of long-chain ceramides in endothelial cells and macrophages (67). In these systems, C16:0 ceramide was reported to activate Gq-mediated signaling, promoting inflammasome activation and vascular inflammation, while genetic or pharmacological inhibition of these receptors attenuated inflammatory responses without altering lipid abundance. These findings introduce a plausible ligand–receptor axis linking ceramides to inflammatory signaling, though its specificity and physiological scope remain to be fully defined.

Similarly, in adipose tissue, the Gi-coupled receptor FPR2 has been reported to bind select ceramide species and suppress thermogenic programs. Structural and functional analyses suggest that FPR2 interacts with C16:0, C18:0, and C20:0 ceramides, leading to reduced intracellular cAMP and blunted UCP1 expression (68). Together, these data support a receptor-dependent mechanism through which ceramides may influence energy expenditure, while leaving open questions regarding tissue selectivity and relative contribution to whole-body metabolic regulation.

Taken together, these studies raise the possibility that ceramides participate in lipid-sensing signaling networks through receptor-mediated mechanisms, extending their influence beyond membrane organization and intracellular signaling. Determining the robustness, context dependence, and physiological relevance of these pathways will be critical for assessing their contribution to metabolic and inflammatory disease.

### Apoptotic thresholds

At higher concentrations, ceramides shift from adaptive stress mediators to triggers of apoptosis (69) (Fig. 5). Elevated ceramide levels promote mitochondrial outer membrane permeabilization, cytochrome c release, and caspase activation—particularly when nutrient excess is sustained or accompanied by additional stressors such as hypoxia, ER stress, or cytokine exposure (70, 71, 72, 73, 74, 75, 76). This terminal response eliminates damaged or irreparably overloaded cells, thereby limiting systemic disruption. While destructive, this function reflects the evolutionary role of ceramides as regulators of cellular integrity in the face of metabolic crisis. They serve not only to buffer and adapt, but to terminate, when adaptation is no longer viable.

Notably, ceramide molecules that comprise a 16-carbon acyl chain play a distinct role in stress signaling by antagonizing insulin signaling, promoting mitochondrial fragmentation and dysfunction, and inducing ER stress (61, 64, 77, 78, 79). Complementing this, Kolesnick and colleagues demonstrated that neutralizing C16:0 ceramide mitigates tissue damage in several disease contexts (80, 81). Together, these findings emphasize that saturated long-chain acyl groups, particularly those comprising a 16-carbon acyl-chain, are active determinants of sphingolipid signaling and stress responsiveness. We also note that the double bond introduced by DES1 is essential for many of ceramide’s signaling functions, as removal of the double bond diminishes its ability to engage in protein interactions, membrane fusion, and stress responses—including the induction of apoptosis (82, 83).

The emphasis on ceramides in this section reflects the disproportionate depth of mechanistic and causal evidence linking this lipid class to nutrient sensing and metabolic regulation. Nonetheless, other sphingolipid species may also influence metabolic physiology, including complex sphingolipids such as glycosphingolipids and sphingomyelins, as well as emerging classes such as deoxyceramides. Glycosphingolipids, in particular, have been implicated in modulation of insulin action, growth factor receptor organization, and cell-cell communication in specific contexts, while sphingomyelins contribute importantly to membrane organization and the biophysical platforms that support signaling. Deoxyceramides have recently attracted attention for their potential metabolic toxicity, though their mechanisms of action remain incompletely defined. Nonetheless, the focus on ceramides here reflects the current strength and maturity of the experimental literature rather than an exclusion of potentially important roles for other sphingolipid classes.

## From cellular sentinels to systemic disease: ceramides in cardiorenal metabolic pathologies

**Fig. 6:**
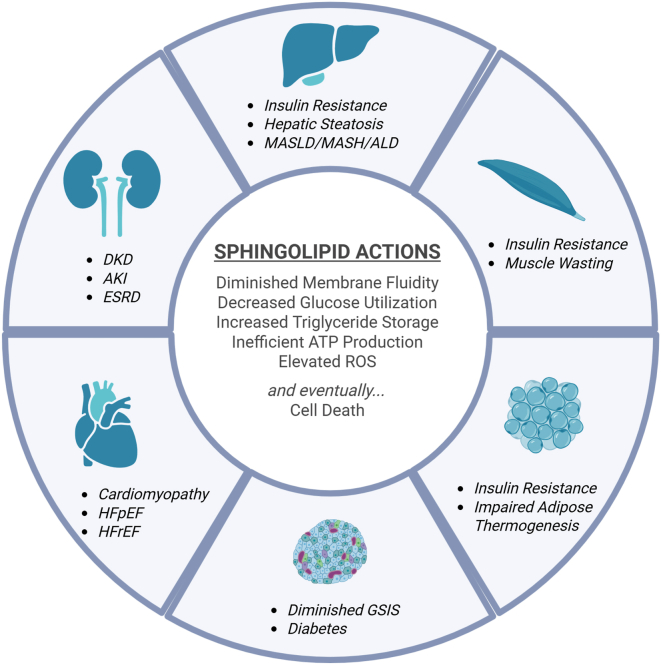
Tissue-specific actions of sphingolipids and their contribution to cardiometabolic disease. Sphingolipids impair metabolic flexibility and mitochondrial function across multiple organs. Central actions include diminished membrane fluidity, impaired glucose utilization, enhanced triglyceride storage, inefficient ATP production, elevated reactive oxygen species (ROS), and—ultimately—cell death. These effects manifest in diverse disease states, including: liver: insulin resistance, hepatic steatosis, and progression to metabolic dysfunction–associated steatotic liver disease (MASLD), metabolic dysfunction–associated steatohepatitis (MASH), and alcoholic liver disease (ALD). Skeletal muscle: insulin resistance and muscle wasting. Adipose tissue: insulin resistance and impaired adipose thermogenesis. Pancreatic islets: diminished glucose-stimulated insulin secretion (GSIS) and diabetes. Heart: lipotoxic cardiomyopathy, including heart failure with preserved ejection fraction (HFpEF) and heart failure with reduced ejection fraction (HFrEF). Kidney: diabetic kidney disease (DKD), acute kidney injury (AKI), and end-stage renal disease (ESRD). Together, these multifaceted actions highlight the pathological reach of sphingolipids in metabolic disease.

The multifaceted cellular actions of ceramides—modulating membrane stability, nutrient handling, mitochondrial efficiency, and ultimately cell survival—manifest in tissue-specific patterns that drive the development and progression of cardiometabolic and renal diseases (Fig. 6). The accumulation of distinct ceramide species in key metabolic organs has been consistently linked to insulin resistance, hepatic steatosis, heart failure, and kidney dysfunction in humans, and interventional studies in rodents provide strong evidence for causality. Below, we examine how ceramide signaling manifests across major tissues to produce systemic metabolic dysfunction.

### Liver: ceramide-induced rewiring of hepatic lipid metabolism

The liver is both a major site of ceramide synthesis and a primary target of its metabolic effects. Hepatic ceramides accumulate in response to excess saturated fat intake, inflammatory cytokines, or farnesoid-X-receptor–mediated intestinal signaling (84, 85, 86). In humans, hepatic ceramide content correlates with steatosis severity, insulin resistance, and MASH (84, 87, 88).

In rodent models, genetic deletion and/or pharmacologic inhibition of ceramide synthetic enzymes—particularly SPT, CERS6, or DES1—lowers hepatic ceramide levels, restores insulin signaling, and reduces TG accumulation (55, 57, 61, 64, 78, 89). Targeted knockout of hepatic DES1 (55) or conditional overexpression of the ASAH1 ceramidase (42, 90), which degrades ceramides in lysosomes, also reverse steatosis, dampen hepatic glucose output, and improve whole-body glycemic control. Similarly, knockout of ceramide biosynthesis in adipose tissue also resolves hepatic steatosis, suggesting the existence of crosstalk mechanisms between the two organs (42, 55).

Mechanistically, ceramides impair hepatic insulin action by inhibiting Akt, thereby promoting gluconeogenesis and inhibiting glycogen synthesis (55). Simultaneously, they drive TG accumulation by promoting SREBP1-mediated TG production and CD36 translocation (42, 55). Lastly, through their effects on MFF, they fragment mitochondria to decrease nutrient oxidation and energy production (64, 78).

Ceramide chain length plays a critical role in hepatic metabolic regulation. Loss of CERS2, the enzyme responsible for generating very long–chain ceramides, disrupts glucose metabolism due to a compensatory increase in CERS6 expression (61, 91). In contrast, CERS6 ablation enhances glucose homeostasis, reduces hepatic steatosis, and improves mitochondrial function (64, 78).

Collectively, these data implicate C16-ceramide in the key tissue abnormalities that underlie metabolic-associated steatotic liver disease/steatohepatitis (MASH) and alcoholic liver disease.

### Adipose tissue: impaired glucose metabolism

Human studies demonstrate that sphingolipid levels—including ceramides—are significantly elevated in the subcutaneous adipose tissue of individuals with type 2 diabetes, independent of body mass index, and correlate with systemic insulin resistance and hepatic steatosis (54, 78, 92, 93). Elevated adipose ceramides impair insulin-stimulated glucose utilization, reduce mitochondrial oxidative function, and inhibit lipolysis (53, 54). These effects are cell-autonomous and particularly disruptive in thermogenic adipocytes, where ceramide accumulation reduces mitochondrial respiration and blunts energy expenditure (53, 54). Mechanistically, ceramides inhibit insulin signaling at the level of AKT to slow glucose utilization (44) and activate MFF to alter mitochondrial morphology and function (64).

Rodent studies further show that manipulation of ceramide metabolism in adipose tissue exerts profound systemic effects. Adipocyte-specific overexpression of ASAH1, the lysosomal enzyme that degrades ceramides, or deletion of SPTLC2, a critical subunit of SPT, enhances insulin sensitivity, decreases inflammation, and improves overall adipose health (42, 54). Deletion of either SPTLC2 or CERS6 from thermogenic beige or brown adipocytes elicits comparable effects, while also increasing energy expenditure (53, 78).

Mechanistic studies in adipocytes show that ceramides inhibit insulin-stimulated glucose uptake by blunting activation of AKT (42, 44, 53, 54, 94, 95) while inhibiting release of fatty acids by inhibiting activation of lipolytic enzymes (53, 54, 55). They were also shown to induce expression of FGF13, a non-secreted member of the fibroblast growth factor family that inhibits mitochondrial content and suppresses metabolic flexibility (96).

Curiously, two groups reported that adipose-specific depletion of SPT subunits produced lipodystrophy, as adipocytes failed to mature appropriately (97, 98). The reason these groups obtained different results from those mentioned above is unclear, but may relate to the timing or magnitude of gene depletion.

### Skeletal muscle: metabolic inflexibility and insulin resistance

Skeletal muscle is a major site of insulin-stimulated glucose disposal and a sensitive target of ceramide-induced metabolic dysfunction. Several studies, conducted in both humans and rodents, reveal that muscle ceramide content—particularly C16:0 and C18:0 species—inversely correlates with insulin-stimulated glucose uptake (99, 100, 101, 102, 103, 104, 105). These observations helped inform the ‘Athlete’s Paradox,’ in which endurance-trained individuals accumulate intramyocellular lipid droplets but maintain high insulin sensitivity—and might be explained by differences in ceramide content or distribution (106). However, the field has been marked by conflicting data, with some groups reporting no ceramide accumulation in insulin resistant muscle (107, 108, 109, 110, 111), leaving the issue controversial and unresolved.

Mechanistically, ceramides impair insulin signaling in myotubes and isolated muscle strips by activating PP2A and atypical protein kinase Cζ, which together inhibit AKT phosphorylation and block GLUT4 translocation (45, 47, 49, 57, 112). Ceramides also impair mitochondrial efficiency and promote ROS production (59, 113, 114, 115, 116, 117), in part by depleting coenzyme Q10 from inner mitochondrial membranes. The induction of ROS may also contribute to the inhibition of GLUT4 translocation and glucose uptake (115). In vivo, global inhibition of ceramide biosynthesis—via pharmacologic agents such as the SPT inhibitor myriocin or the DES1 inhibitor fenretinide—restores insulin sensitivity, improves mitochondrial function, and enhances glucose oxidation in obese or aged rodents (56, 57, 89, 118). Another SPT inhibitor, ALT-007, was shown to potently improve muscle mass and function in a mouse model of sarcopenia (119).

Tissue-specific interventions have focused on CERS1, the major isoform in skeletal muscle and the only CERS that exclusively adds 18 carbon acyl-chains to the sphingoid backbone. Ablation of CERS1, either in the whole body or selectively in skeletal muscle, reduced C18-ceramides and improved glucose homeostasis (120). Curiously, the CERS1 inhibitor P053, which targets the muscle-specific (dihydro)ceramide synthase, improved muscle fat oxidation, without improving overall insulin sensitivity (121).

Notably, work from the Watt lab suggests that some muscle ceramides may be delivered via liver-derived lipoproteins, rather than being synthesized locally (122). These data are consistent with findings using other genetic knockouts, where whole-body or liver-targeted ablation of DES1 or CERS6 improved muscle insulin sensitivity (55, 78), but muscle-specific depletion of CERS6 did not (120). These findings highlight the importance of blood ceramides and their trafficking between tissues as a mechanism of obesity-linked muscle dysfunction.

### Heart: Contractile dysfunction and energetic Inefficiency

Cardiomyocytes are especially vulnerable to ceramide accumulation due to their high energy demands and limited capacity for lipid storage. In humans, plasma ceramide levels correlate with heart failure severity, left ventricular hypertrophy, and hospitalization risk. Specific ceramide subspecies—particularly C16:0, C18:0, and C24:1—are predictive of adverse cardiac events (123, 124, 125, 126, 127, 128, 129, 130) and have been incorporated into a prognostic diagnostic that is in clinical use (131, 132). Recent studies with human heart biopsies reveal that ceramides also accumulate in the failing myocardium (133).

Studies in preclinical models reveal that cardiac ceramide accumulation perturbs mitochondrial energetics, disrupts glucose metabolism, exacerbates fibrosis, and induces apoptosis (133, 134, 135, 136, 137, 138), exacerbating arrhythmic risk and impairing cardiac reserve. Genetic or pharmacological suppression of ceramide synthesis preserves mitochondrial function, improves ejection fraction, and limits fibrosis under pressure overload or ischemic stress (52, 133, 139). Moreover, a zinc finger-containing transcription factor termed kruppel-like factor 5 was shown to drive de novo ceramide biosynthesis to promote ischemic cardiomyopathy (140). Lastly, di Lorenzo and colleagues achieved cardiomyocyte-selective ablation of the regulatory protein Nogo-A, which increased SPT complex activity and exacerbated heart failure caused by transverse aortic constriction (141). These findings position ceramides as active participants in the pathogenesis of several cardiac pathologies.

Mechanistically, Cowart and colleagues demonstrated that very long-chain ceramides contribute to mitochondrial dysfunction, oxidative stress, and cell death in cultured cardiomyocytes (142). They also identified a potential role for ceramides comprising a shorter sphingoid base, which are produced by SPTLC3, to the mitochondrial impairments that underlie ischemic cardiomyopathy (63). They demonstrated that the atypical ceramides associate with complex I of the electron transport chain to alter subunit composition and mitochondrial efficiency (63). Schulze and colleagues conducted molecular studies in human inducible pluripotent cardiomyocytes, finding that ceramides inhibit AKT (via PP2A) and induced various markers of oxidative stress (143).

Collectively, these studies show strong suggestive evidence that ceramides contribute to cardiac dysfunction, including exciting data that blood ceramides correlate with cardiac dysfunction and that whole-body ceramide depletion normalizes cardiac function. However, our understanding of ceramide mechanisms would benefit from additional studies probing the consequences of tissue-specific interventions, including detailed molecular analyses.

### Pancreatic β-cells: ceramides and the failure of insulin secretion

Pancreatic β-cells are highly sensitive to lipid-induced stress, and ceramides have emerged as central mediators of glucolipotoxicity. Ceramides disrupt β-cell function by inhibiting insulin gene transcription (144, 145) and mitochondrial respiration (146, 147), inducing ER stress (148), and promoting apoptosis (149, 150). Moreover, Kowlura and colleagues reported that ceramides modulate the small GTP-binding protein Ras-related C3 botulinum toxin substrate 1 (RAC1) (147, 151, 152) and the atypical PP2A subunit ALPHA4 (153) to exacerbate stress responses and impair glucose-stimulated insulin secretion. Recent findings suggest that ceramide chain length may influence outcome, with very long chain ceramides (e.g., C24:0) supporting insulin processing through cis-Golgi maturation (154, 155), while C16:0 species exert the aforementioned cytotoxic effects.

Rodent studies support a causal role for ceramides in β-cell failure. The Unger laboratory first demonstrated that ceramides accumulated in palmitate-treated islets and induced apoptosis, and that inhibitors of de novo ceramide synthesis—such as fumonisin B1 and L-cycloserine—prevented this effect both ex vivo and in vivo (156, 157). Subsequent studies in immortalized INS-1 and MIN6 cells confirmed that palmitate’s ability to trigger β-cell apoptosis and impair insulin secretion are ceramide-dependent (149, 150). Genetic ablation of CerS2 in mice—which leads to reductions in the protective very-long-chain ceramides and induction of the deleterious long-chain C16 ceramides—reduced insulin maturation and compromised glucose control (154, 155).

Collectively, these findings suggest that ceramides, particularly the long-chain ceramides, serve as critical regulators of β-cell survival and function.

### Kidney: ceramides as drivers of diabetic nephropathy

Ceramides are increasingly recognized as key mediators of kidney injury across both chronic proteinuric diseases and acute tubular insults. In proteinuric disorders such as diabetic nephropathy, ceramides accumulate within glomeruli and renal tubules, where they disrupt mitochondrial function, promote inflammatory signaling, and trigger cell death—driving proteinuria, glomerulosclerosis, and progressive loss of renal function (65). Recent work has identified CERS6 as a central contributor to this pathology: it is enriched in podocytes, upregulated in diabetic and toxic nephropathy models, and sufficient to induce proteinuria when overexpressed (158). Mechanistically, CerS6-derived C16:0 ceramide binds VDAC1 on the mitochondrial outer membrane, promoting mitochondrial DNA leakage and activation of the cGAS–STING pathway, thereby amplifying immune–inflammatory injury.

Complementing these glomerular mechanisms, acute kidney injury (AKI) is now recognized to involve a parallel, ceramide-driven program centered in proximal tubule epithelial cells (159). Ischemic and toxic AKI induce de novo ceramide synthesis within tubules, with preferential accumulation of long-chain C16–C18 species that disrupt mitochondrial cristae organization, respiratory supercomplex assembly, and oxidative phosphorylation. Genetic or pharmacologic inhibition of DES1 preserves mitochondrial architecture, attenuates tubular injury, and maintains renal function in experimental AKI, while urinary ceramides track closely with injury severity in human cohorts.

Together, these findings position ceramide signaling as a unifying driver of renal injury across glomerular and tubular compartments and highlight multiple nodes within the ceramide biosynthetic pathway as promising therapeutic targets in both acute and chronic kidney disease.

## Therapeutic potential and the future of ceramide-targeted interventions

As the evidence linking ceramides to cardiometabolic and renal disease continues to mount, a new frontier is emerging—one in which sphingolipid metabolism may be harnessed for therapeutic benefit. Much like LDL cholesterol in the mid-20th century, ceramides have transitioned from a biochemical curiosity to a validated risk marker, and now stand on the cusp of becoming both a diagnostic tool and a modifiable target.

Multiple clinical studies have established that circulating ceramides—particularly species such as C16:0, C18:0, and C24:1—predict cardiovascular events, heart failure hospitalizations, and renal decline with a precision rivaling or exceeding traditional lipid measures. These insights have led to the development of ceramide-based risk scores, now used in select preventive cardiology clinics to stratify risk and guide treatment intensity. Importantly, ceramide levels often rise before overt metabolic disease, offering a window for early intervention.

Therapeutically, several strategies to lower or reprogram ceramide synthesis are under investigation. Small-molecule inhibitors of SPT and ceramide synthases have shown efficacy in preclinical models, reversing insulin resistance, hepatic steatosis, and cardiac dysfunction. DES1 is another intriguing therapeutic target that shows an attractive combination of efficacy and safety (82). Enhancement of ceramide degradation through activation of ceramidases—either pharmacologically or via adiponectin receptor agonists—offers a complementary approach. Because sphingolipids play essential roles in membrane structure, development, and cell survival, these strategies will require careful attention to dose, selectivity, tissue targeting, and long-term safety.

The translational implications are profound: if ceramides can be lowered safely, and if their reduction leads to meaningful clinical benefit—as suggested by rodent studies and early human data—then routine screening for ceramides could enable primary prevention in high-risk populations. Much as statins reshaped cardiovascular care by intervening on cholesterol pathways before the onset of atherosclerosis, ceramide-directed therapies may allow us to intercept metabolic disease at its earliest molecular stages—not merely reacting to disease, but preempting it.

Moving forward, key challenges will include optimizing pharmacologic selectivity, ensuring long-term safety, and identifying the subpopulations most likely to benefit. Yet the path forward is conceptually clear: by treating ceramides as both sentinel signals and modifiable mediators, we may be entering an era where lipid biology once again transforms chronic disease prevention.

While much of the discussion above emphasizes the pathological consequences of ceramide accumulation under conditions of chronic nutrient excess, it is important to note that sphingolipids are indispensable components of normal cellular physiology. Beyond their structural roles in membranes, sphingolipids support neuronal survival, immune signaling, epithelial barrier function, and development. Accordingly, complete or sustained suppression of sphingolipid synthesis can be deleterious, and the biological impact of ceramides is highly dependent on dose, duration, tissue context, and subcellular localization. These considerations are central to evaluating therapeutic strategies aimed at modulating sphingolipid metabolism.

## Conclusion

Across diverse tissues, ceramides translate metabolic surplus into organ dysfunction. They act not only as markers of lipid excess but as active effectors that reshape membrane properties, suppress metabolic flexibility, and enforce cellular limits through mitochondrial reprogramming and apoptotic commitment. Their accumulation across liver, adipose, muscle, heart, β-cells, and kidney creates a systemic axis of lipotoxic stress that underpins the progression of a wide range of cardiometabolic and renal diseases.

By connecting nutrient state to cellular fate, ceramides serve as both sentinels and sculptors of metabolic health. Their predictive value in human disease and the striking therapeutic efficacy of ceramide-lowering interventions in rodents position them as actionable targets in the prevention and treatment of diabetes, heart failure, and chronic kidney disease.

## Declaration of Generative AI and AI-Assisted Technologies in the Writing Process

ChatGPT-4o and Gemini were used to improve the readability and language of the manuscript. All content was subsequently reviewed and edited by the author, who takes full responsibility for the final version.

## Conflict of interest

Scott Summers is a consultant and shareholder with Centaurus Therapeutics.
